# Food security status and cardiometabolic health among pregnant women in the United States

**DOI:** 10.3389/fgwh.2023.1286142

**Published:** 2024-02-13

**Authors:** Jamie A. Murkey, Symielle A. Gaston, Christopher W. Payne, W. Braxton Jackson, Chandra L. Jackson

**Affiliations:** ^1^ Epidemiology Branch, National Institute of Environmental Health Sciences, National Institutes of Health, Department of Health and Human Services, Research Triangle Park, NC, United States; ^2^ Social & Scientific Systems, Inc., a DLH Holdings Company, Durham, NC, United States; ^3^ Intramural Program, National Institute on Minority Health and Health Disparities, National Institutes of Health, Department of Health and Human Services, Bethesda, MD, United States

**Keywords:** food insecurity, ideal cardiovascular health, cardiovascular disease, health inequities, pregnant women, race factors, social determinants of health

## Abstract

**Introduction:**

Pregnant women and their offspring are particularly vulnerable to food insecurity and its adverse effects during critical periods of fetal development. Racially/ethnically minoritized women in the United States (US) who are pregnant are additionally burdened by food insecurity, which may exacerbate cardiovascular health (CVH) disparities. Despite heightened social vulnerability, few studies have employed an intersectional framework, including race and gender, to assess the food insecurity and CVH relationship.

**Methods:**

We used 2012–2018 and 2020 National Health Interview Survey data among US pregnant women aged 18–49 years old (*N* = 1,999) to assess the prevalence of food insecurity status by race/ethnicity and to investigate household food security status in relation to ideal CVH, using a modified ideal CVH (mICVH) metric. We categorized food security status as “very low/low”, “marginal”, or “high”. To assess mICVH, a summary score of 7 clinical characteristics and health behaviors was dichotomized as yes [(7)] vs. no [<7]. Prevalence ratios (PRs) and 95% confidence intervals (CIs) of associations between food security status and mICVH were estimated using Poisson regression with robust variance. Models were adjusted for age, household income, educational attainment, geographic region, marital status, alcohol consumption, survey year, and race/ethnicity (in overall model).

**Results:**

The mean age ± standard error was 29.0 ± 0.2 years. Among pregnant women, 12.7% reported “very low/low”, 10.6% reported “marginal”, and 76.7% reported “high” food security. “Very low/low” food security prevalence was higher among NH-Black (16.2%) and Hispanic/Latina (15.2%) pregnant women compared to NH-White (10.3%) and NH-Asian (3.2%) pregnant women. The mICVH prevalence was 11.6% overall and 14.5% for NH-White, 4.1% for NH-Black, 5.0% for Hispanic/Latina, and 26.7% for NH-Asian pregnant women. Among all pregnant women, “very low/low” and “marginal” vs. “high” food security status was associated with a lower prevalence of mICVH {[PR_very low/low_ = 0.26 (95% CI: 0.08–0.75)]; [PR_marginal _= 0.47 (95% CI: 0.23 −0.96)]}.

**Conclusion:**

Household food insecurity was higher among pregnant women in minoritized racial/ethnic groups and was associated with lower mICVH prevalence. Given the higher burden of food insecurity among minoritized racial/ethnic groups, food security may be an important intervention target to help address disparities in poor CVH among pregnant women.

## Introduction

Food insecurity, defined as a lack of access to nutritious substances because of financial or resource constraints, is a major public health challenge that is associated with poor cardiovascular health (CVH) (1–3). Prior literature suggests that food insecurity is associated with cardiovascular disease (CVD) morbidity and mortality risk (2–6). Vulnerable groups are disproportionately impacted by food insecurity. For instance, pregnant women and their offspring are particularly vulnerable to food insecurity and its adverse effects during critical periods of fetal and child development (7, 8). Food insecurity during pregnancy can compromise fetal development (e.g., spina bifida due to inadequate dietary intake of folic acid) (7) as well as contribute to low birth weight (9) and preterm birth (8), all of which have been disproportionately observed among the offspring of NH-Black women (10–12). Pregnancy can also alter cardiovascular functioning (13), leading to poor CVH, which disproportionately burdens pregnant and postpartum women from minoritized racial/ethnic groups (14, 15). Additionally, women from racially/ethnically minoritized groups in the United States (US) are burdened by food insecurity (1, 16–19), which may consequently exacerbate existing CVH disparities among pregnant women (14, 15). Food insecurity is projected to worsen as climate change increasingly disrupts food systems, potentially reducing the accessibility and affordability of food available to vulnerable groups (20, 21). Food insecurity may also be facilitated by the neighborhood environment of pregnant women, ultimately influencing health behaviors. For instance, structurally racist practices, such as redlining, has symbiotically driven the disinvestment of communities while simultaneously giving rise to food deserts (areas lacking healthy food options) and food swamps (areas heavily concentrated with unhealthy food options), limiting access to nutrient dense food options for pregnant women (22–25). It is worth noting that the term food apartheid (inequitable food environments stremming from racist structures and practices) has been recommended to be used in place of “food deserts” (26, 27).

In 2022, the American Heart Association (AHA) introduced the *Life's Essential 8* as an updated public health strategy to quantify population-level ideal CVH and guide CVD risk mitigation (28). Consisting of modifiable health behavior and CVD risk factors, AHA's *Life's Essential 8* includes smoking status, body mass index (BMI), physical activity, diet, total cholesterol, blood pressure, fasting glucose, and sleep duration, which is a recently established risk factor for CVD (28, 29). Prior studies suggest that compared to men, women are more likely to be food insecure and have a lower prevalence of ideal CVH (1, 18, 19, 30, 31). Additionally, one US study reported that non-Hispanic (NH)-White adults were three times more likely to have ideal CVH compared to NH-Black and Hispanic/Latinx adults (32). Thus, pregnant women from minoritized racial/ethnic groups are more likely to have a lower ideal CVH prevalence compared to those who are NH-White, potentially increasing maternal morbidity risk.

Few studies have employed an intersectional framework—predicated on the idea that the interconnection of systems of power (e.g., race, ethnicity, gender, socioeconomic status) shape oppression and privilege (33)—while investigating the food insecurity and CVH relationship. Fewer were nationally-representative and included pregnant women from minoritized racial/ethnic groups, despite their heightened social vulnerability. Therefore, we investigated household food security status in relation to mICVH prevalence among pregnant women in the US. Since racial/ethnic disparities are observed among the general population for food insecurity (1, 16–19) and mICVH prevalence (32, 34), we hypothesized that “very low/low” and “marginal” food security prevalence as well as mICVH prevalence would be higher among pregnant women belonging to minoritized racial/ethnic groups compared to NH-White women. We also hypothesized that “very low”/'low' and “marginal” vs. “high” food security status is associated with lower mICVH prevalence among pregnant women.

## Methods

### Study population

We used 2012–2018 and 2020 National Health Interview Survey (NHIS) serial cross-sectional data, which uses three-stage cluster probability sampling to survey non-institutionalized individuals within US households. Further details on the NHIS study design and recruitment have been previously described (35). All NHIS participants provided written informed consent. Additionally, the National Institute of Environmental Health Sciences Institutional Review Board waived approval for the use of non-identifiable, publicly available NHIS data. The final response rate among sampled adults was 50.6% [range: 61.2% (2012)—45.2% (2020)]. Notably, lower average response rates in 2012 compared to 2020 are likely attributed to the shift from in-person to telephone-only household interviews conducted during the COVID-19 pandemic, resulting in lower-income households being underrepresented in the 2020 study sample (36).

### Exposure assessment: Food security status

We defined household food security status as “very low/low”, “marginal”, or “high” using the validated US Department of Agriculture (USDA) Family Food Supplement scale. Our study included the 10-item Family Food Supplement, which was derived from the 18-item Food Security Survey Module (37). The 18-item Food Security Survey Module has been shown to have good reliability (Cronbach *α* = 0.81 for households with children and 0.74 for all households) (38). Participants were asked about food availability and consumption in the past 30 days. For example, questions included “How often (often true; sometimes true; never true; or don't know) did the following happen in the past 30 days”: “We couldn't afford to eat balanced meals; We worried whether our food would run out before we got money to buy more; We couldn't’ afford to eat balanced meal”. Participants were also asked whether or not (yes or no) any of the followed occurred during the past 30 days: “Did any of your family not eat for a whole day because there wasn't enough money for food?”; Did you ever cut the size of meals or skip meals because there wasn't enough money for food?”; Did you ever eat less than you felt you should because there wasn't enough money for food?”. A complete list of the questions is summarized in Supplementary Table S2. If participants responded to an item affirmatively as “yes”, “often true”, or “sometimes true”, responses were counted as 1 and otherwise as 0 (37). Responses were then summed (0–10) and categorized as “very low/low” (3–10), “marginal” (1–2), and “high” (0) food security (37).

### Outcome assessment: Ideal cardiovascular health

Modeled after the AHA's *Life's Essential 8*, we developed a modified version of the ideal CVH metric—mICVH—since diet data is unavailable in NHIS (28). A summary score of 7 self-reported clinical characteristics and health behaviors were dichotomized (yes [(7)] or no [<7]) using the following indicators, which were assigned a value of 1 if present and a value of 0 if absent: (1) smoking status (never smoked/quit smoking >12 months prior to study enrollment); (2) recommended body mass index (≥18.5 kg/m^2^–<25 kg/m^2^); (3) meet physical activity guidelines for Americans [≥150–300 min/week moderate exercise or ≥75–150 min/week vigorous exercise(39)]; (4) recommended sleep duration (7–9 h per night); and no prior diagnosis of (5) dyslipidemia, (6) hypertension, or 7) prediabetes/diabetes. Therefore, if participants indicated “yes” for each indicator, they were considered to have mICVH.

### Potential confounders

We considered potential sociodemographic and lifestyle confounders *a priori* based on prior literature. Sociodemographic confounders included: age (18–30 or 31–49 years); annual household income (<$35,000, $35,000–$74,999, ≥$75,000); marital status (married/cohabitating, single/no live-in partner, or divorced/separated/widowed); educational attainment (<high school, high school graduate, some college, or ≥college); geographical region of residence (Northeast, Midwest, South, or West); and survey year. Alcohol consumption [current (heavy), current (≤moderate), former, or lifetime abstainer] was considered as a lifestyle confounder.

### Potential modifiers

Race/ethnicity (NH-Asian, Hispanic/Latinx, NH-Black or NH-White) was investigated as a potential effect modifier based on prior literature revealing lower food insecurity and high cardiovascular disease prevalence among women from minoritized racial/ethnic groups (1, 17, 19). In the NHIS, participants self-identify race and ethnicity using standard categories defined by the post 1997 Executive Office of the President, Office of Management and Budget (40). Pregnant women identifying as races and ethnicities other than NH-White, NH-Black, Hispanic/Latina, and NH-Asian [e.g., American Indian/Alaska Native, Native Hawaiian/Pacific Islander, multiracial] were described as “NH-Other” due to small sample sizes and heterogeneity if groups were combined.

### Statistical analyses

Among women ≥18 years of age who participated in the 2012-2018 and 2020 NHIS (*n* = 140,817), we excluded women ≥50 years of age (*n* = 74,313) and those who did not identify as a pregnant (*n* = 64,441). Further, women were excluded if they were missing data on food security status, mICVH metrics [fasting glucose, blood pressure, cholesterol, dietary patterns, physical activity, body mass index (BMI) and smoking status], pregnancy status, as well as the following confounders: age, sex/gender, or race/ethnicity (*n* = 124). After applying these exclusion criteria, the final analytic sample was 1,999 participants.

Data were weighted to obtain nationally representative estimates. We reported mean ± standard error for age, along with weighted percentages (to account for the complex survey design) for sociodemographic, lifestyle, health behavior, and clinical factors in the overall population and by household food security status. Weighted Poisson regression models with robust variance were used to estimate prevalence ratios (PRs) and 95% confidence intervals (CIs) of associations between food security status category and mICVH overall and by race and ethnicity. We report unadjusted and adjusted models for age, annual household income, educational attainment, geographic region, marital status, alcohol consumption, survey year, and race/ethnicity (in overall model). In models, “high” food security status was used as the reference group to compare “low/very low” and “marginal” food security status. We investigated potential effect modification/differences in associations between food security status and mICVH by including a multiplicative interaction term (race/ethnicity*food security status) in the overall model and performed a Wald test of the interaction term. A two-sided alpha level of 0.05 was used to determine statistical significance in all analyses. All analyses were conducted using survey procedures in Stata version 15.1 (StataCorp, LLC, College Station, TX).

## Results

### Study population characteristics

Among the 1,999 included participants, the mean age ± standard error was 29.0 ± 0.2 years (Table 1). Food security status prevalence was 12% for “very low/low”, 9.0% for “marginal”, and 79% for “high”. “Very low/low” food security prevalence was higher among pregnant women identifying as NH-Black (16.2%), and Hispanic/Latina (15.2%) compared to both NH-White (10.3%) and NH-Asian (3.2%) pregnant women (Table 2). The mICVH prevalence was 11.6% overall and 14.5% for NH-White, 4.1% for NH-Black, 5.0% for Hispanic/Latina, 26.7% for NH-Asian pregnant women, and 6.1% for pregnant women identifying as races and ethnicities other than NH-White, NH-Black, Hispanic/Latina, or NH-Asian (Supplementary Figure S1). Pregnant women with “very low/low” food security had the highest prevalence of <high school educational attainment (18.5%) as well as annual household income <$35,000 (70.7%), were the least likely to be married/living with a partner/cohabitating (52.6%), and largely resided in the Southern region of the US (45.2%) (Table 1). Further, pregnant women with “very low/low food security” had the highest prevalence of current smoking (22.2%), current alcohol consumption (≥1 drink in the past year: 52.8%), and the lowest prevalence of excellent/very good/good health status (87.7%) as well as mICVH (1.6%) (Table 3).

**Table 1:** Sociodemographic characteristics among pregnant adults aged 18–49 years old by food security status, National Health Interview Survey, 2012–2018, 2020, (*N*=1,999).

Characteristics^a^	Food security status			
	Very Low/Low *n *= 254 (12.0%)^a^	Marginal *n *= 212 (9.0%)^a^	High *n *= 1,533 (79.0%)^a^	Overall *n *= 1,999 (100%)
Sociodemographic				
Age, mean ±SE (years)	27.5 ±.51	27.8 ±.57	29.3 ±.18	29.0 ±.16
18–30	72.0	72.5	59.5	62.2
31–49	28.0	27.5	40.5	37.8
Race/ethnicity				
Hispanic/Latinx	22.8	24.4	16.5	17.9
NH-Asian	1.3	4.9	5.5	4.9
NH-Black	21.2	23.8	14.0	15.7
NH-Other	4.2	4.8	1.9	2.5
NH-White	50.5	42.1	62.2	59.0
Educational Attainment^b^				
< High School	18.5	13.0	6.9	8.8
High School graduate	38.6	38.6	20.4	24.1
Some College	32.9	36.0	29.6	30.6
≥ College	10.0	12.4	43.1	36.5
Annual household income^b^				
<$35,000	70.7	51.9	25.4	33.1
$35–$74,999	19.8	39.6	30.2	29.8
≥$75,000	9.4	8.5	44.5	37.1
Unemployed/not in labor force^b^	60.9	57.7	34.7	39.9
Marital status^b^				
Married/living with partner/cohabitating	52.6	68.6	80.5	76.1
Divorced/widowed	16.9	5.1	4.6	6.1
Single/no live-in partner	30.5	26.3	14.8	17.8
Region of residence				
Northeast	15.5	19.7	15.5	15.9
Midwest	23.1	16.6	22.5	22.0
South	45.2	39.0	39.9	40.5
West	16.2	24.8	22.1	21.6

SE, standard error; NH, non-Hispanic.

^a^ Note all estimates are weighted for the survey's complex sampling design. Percentage may not sum to 100 due to missing values or rounding.

^b^ Participants were missing information for educational attainment, annual household income, and marital status

**Table 2:** Prevalence ratios of modified ideal cardiovascular health among pregnant adults who reported experiencing very/low and marginal food security compared to high food security overall, and by race and ethnicity^a^, National Health Interview Survey, 2012–2018, 2020, (*N* = 1,999).

	Food security status, %	mICVH, %	Prevalence ratio (95% confidence interval) ^b^	
			Crude	Adjusted
Overall (*N* = 1,999)		11.6		
High	79.0	14.0	Referent	Referent
Marginal	9.0	3.9	**0.28** **(0.14, 0.56)**	**0.47** **(0.23, 0.96)**
Very low/low	12.0	1.6	**0.11** **(0.04, 0.32)**	**0.24** **(0.08, 0.75)**
Hispanic Latinx (*n* = 437)		5.0		
High	72.5	6.2	Referent	Referent
Marginal	12.2	3.3	0.54 (0.14, 2.06)	0.41 (0.10, 1.67)
Very low/low	15.2	0.8	0.13 (0.02, 0.97)	NE
NH-Asian (*n* = 121)		26.7		
High	87.8	29.8	Referent	Referent
Marginal	9.0	6.0	NE	NE
Very low/low	3.2	0.0	NE	NE
NH-Black/African American (*n* = 278)		4.1		
High	70.2	4.7	Referent	Referent
Marginal	13.6	3.6	0.77 (0.14, 4.09)	3.77 (0.43, 33.1)
Very low/low	16.2	1.9	0.40 (0.05, 3.33)	0.14 (0.01, 1.38)
NH-White (*n* = 1,103)		14.5		
High	83.3	16.9	Referent	Referent
Marginal	6.4	4.3	**0.25** **(0.08, 0.80)**	0.34 (0.10, 1.08)
Very low/low	10.3	2.0	**0.12** **(0.03, 0.48)**	**0.26** **(0.07, 0.98)**

mICVH, modified ideal cardiovascular health; NH, non-Hispanic; NE, not able to estimate.

Bolded values indicate statistical significance at a two-sided *p*-value <0.05.

Models are adjusted for age (18–30 years, 31–49 years), annual household income (<$35,000, $35,000–$74,999, ≥$75,000), marital status (married/cohabitating, single/no live-in partner, divorced/separated/widowed), educational attainment (<high school, high school graduate, some college, ≥college), region of residence (Northeast, Midwest, South, West), alcohol consumption [current (heavy), current ( ≤ moderate), former, lifetime abstainer], and survey year. Models in the total/overall sample are additionally adjusted for race and ethnicity (Hispanic/Latinx, NH-Asian, NH-Black/African American, NH-White).

All estimates are weighted for the complex survey design. Bolded values indicate statistical significance at a two-sided *p*-value < 0.05.

^a^ There was a significant Wald test for interaction between race/ethnicity and food security status on modified ideal CVH (*p* < 0.001). Stratified results were inestimable for some race and ethnic groups due to small sample sizes.

^b^ Modified ideal cardiovascular health includes never smoking/quit >12 months prior to interview, BMI 18.5 − < 25 kg/m^2^, meeting physical activity guidelines, sleep duration of 7–9 h, and no dyslipidemia, hypertension, or prediabetes/type 2 diabetes.

**Table 3:** Health behavior and clinical characteristics among pregnant adults aged 18–49 years old by food security status, National Health Interview Survey, 2012–2018, 2020, (*N* = 1,999).

Characteristics^a^	Food security status			
	Very low/Low *n *= 254 (12.0%)^a^	Marginal *n *= 212 (9.0%)^a^	High *n *= 1,533 (79.0%)^a^	Overall *n *= 1,999 (100%)
Health behaviors				
Smoking status				
Never/quit >12 months prior	67.4	84.0	86.4	83.9
Former/quit ≤12 months ago	10.5	7.2	6.6	7.1
Current	22.2	8.9	7.1	9.0
Alcohol consumption^b^				
Lifetime abstinence (<12 drinks in life)	28.3	32.0	22.5	24.0
Former (no drinks past year)	18.9	20.1	18.1	18.4
Current (≥1 drink past year)	52.8	48.0	59.4	57.6
Leisure-time physical activity (PA)				
Never/unable	53.1	43.2	31.5	35.2
Does not meet PA guidelines	18.3	23.6	26.9	25.6
Meets PA guidelines^c^	28.6	33.2	41.6	39.3
Usual sleep duration				
Very short sleep (<6 h)	13.4	10.2	5.4	6.8
Short sleep (<7 h)	36.0	31.6	20.0	23.0
Recommended (7–9 h)	58.5	59.7	74.3	71.1
Long sleep (>9 h)	5.5	8.7	5.7	5.9
Clinical Characteristics				
Health status				
Excellent/very good/good	87.7	93.5	97.0	95.6
Fair/poor	12.3	6.5	3.0	4.4
Body Mass Index (BMI)				
Underweight (<18.5 km/m^2^)	5.2	3.0	1.2	1.8
Recommended (18.5–<25 kg/m^2^)	28.6	26.9	42.3	39.3
Overweight (25–29.9 kg/m^2^)	22.1	21.7	27.6	26.4
Obesity (>30 kg/m^2^)	44.1	48.4	29.0	32.5
Dyslipidemia^d^	5.2	2.2	2.1	2.5
Hypertension^e^	9.7	11.9	7.9	8.4
Diabetes/prediabetes^f^	7.6	5.5	3.5	4.2
Modified ideal cardiovascular health^g^	1.6	3.9	14.0	11.6

SE, standard error; NH, non-Hispanic.

^a^ Note all estimates are weighted for the survey's complex sampling design. Percentage may not sum to 100 due to missing values or rounding.

^b^ Participants were missing information for alcohol consumption.

^c^ Meets PA guidelines defined as ≥150 min/week of moderate intensity or ≥75 min/week of vigorous intensity or ≥150 min/week of moderate and vigorous intensity.

^d^ Dyslipidemia defined as currently taking prescribed medicine to lower cholesterol high cholesterol in the 12 months prior to interview.

^e^ Hypertension defined as ever told on two or more different visits that you have hypertension or high blood pressure or currently taking prescribed medicine to lower blood pressure.

^f^ Prediabetes defined as ever told by a doctor had prediabetic condition, prediabetes, or borderline diabetes. Type 2 diabetes defined as ever told by a doctor or health professional that you have diabetes or sugar diabetes and being told you have type 2 diabetes.

^g^ Ideal cardiovascular health includes never smoking/quit >12 months prior to interview, BMI 18.5–<25 kg/m^2^, meeting physical activity guidelines, sleep duration of 7–9 h, and no dyslipidemia, hypertension, or prediabetes/type 2 diabetes.

### Food security status and Ideal cardiovascular health

Among pregnant women with “high” food security status, mICVH prevalence was 14%, overall, was highest among pregnant women who identified as NH-Asian (29.8%), and was lowest among NH-Black (4.7%) pregnant women (Table 2). Among all pregnant women, “very low/low” vs. “high” food security status was associated with a 76% lower prevalence of mICVH [PR = 0.24 (95% CI: 0.08–0.75)]. “Marginal” vs. “high” food security status was associated with a 53% lower prevalence of mICVH [PR = 0.47 (95% CI: 0.23–0.96). Although effect measure modification was present (*p*-interaction < 0.001), stratified results were inestimable for some racial and ethnic groups due to small sample sizes. Among NH-White pregnant women, “very low/low” vs. “high” food security status was associated with a lower mICVH prevalence [PR = 0.26 (95% CI: 0.07–0.98)]. Small sample sizes precluded our ability to compare associations between food security status and mICVH for each race/ethnicity included in our study sample.

## Discussion

In this nationally representative study among a racially/ethnically diverse sample of pregnant women, we investigated food security status in relation to mICVH prevalence. We observed racial/ethnic inequities in food insecurity with “very low/low” food security prevalence being higher among Hispanic/Latinx, NH-Black, and NH-Other pregnant women compared to NH-White and NH-Asian pregnant women. We found, in adjusted models, that “very low/low” vs. “high” food security status was associated with a lower prevalence of mICVH, which aligned with our hypothesis. Similarly, “marginal” vs. “high” food security status was also associated with a lower prevalence of mICVH. Estimates for pregnant women from minoritized racial/ethnic groups had wide confidence intervals or could not be estimated due to small sample sizes. However, despite limited power to detect associations by each race/ethnicity included in our study, there was a suggestion that associations between “very low/low” and “marginal” vs. “high” food security status and lower mICVH prevalence would be the strongest for pregnant women from minoritized racial/ethnic groups. It is worth noting that the relative difference between “very low/low”, “marginal”, and “high” food security status is small among NH-Asian pregnant women. Public health impact is likely the largest among pregnant women from minoritized racial/ethnic groups, compared NH-White pregnant women, due to the high burden of low food security and mICVH prevalence, even if the relative associations are the same (41). Food security may be an important intervention target for addressing CVH disparities among pregnant women.

Our findings are consistent with prior studies reporting that food insecurity and low mICVH prevalence among women from minoritized racial/ethnic groups was higher compared to as NH-White women (1, 18, 19, 28, 30–32, 42). Given the importance of nutrition in shaping maternal and fetal outcomes, food insecurity threatens to widen disparities among women from racially/ethnically minoritized groups. For instance, the offspring of NH-Black women experience the greatest burden of low birth weight as well as preterm births, which can be exacerbated by inadequate dietary intake due to food insecurity (7–12). The results of this study also indicate that mICVH prevalence, an independent predictor of CVD risk (43), was the lowest among pregnant women from minoritized racial/ethnic groups (except NH-Asian pregnant women). Disparities in mICVH prevalence, combined with inequities in health conditions experiences during pregnancy [e.g., preeclampsia (44, 45), gestational diabetes (46)], may further exacerbate disparities in CVD risk among pregnant women from minoritized racial/ethnic groups. Without public health interventions implemented to mitigate such inequities in maternal nutrition (e.g., addressing food insecurity), racial/ethnic disparities in poor birth outcomes will persist.

Although understudied, investigating social determinants, shaped by structural inequities, may help researchers better understand mechanisms driving racial/ethnic disparities in poor maternal health outcomes. For instance, access to quality healthcare during prenatal and postpartum periods is crucial for ensuring that the mother and her offspring are healthy. In fact, prenatal and postpartum healthcare settings may help to identify and address food insecurity during pregnancy (47). Further, some healthcare-based interventions (e.g., using a produce prescription program, providing produce vouchers, group prenatal care) (48–51) have been used to target food insecurity and improve cardiometabolic health (52) during pregnancy (48–53). While economic disadvantage may affect utilization of prenatal health care, some literature suggests that racialized pregnancy stigma experienced by women from minoritized racial/ethnic groups in the US can also result in poorer quality of health care during pregnancy and postpartum (54, 55). Other structural inequities contributing to neighborhood environments also contribute to food insecurity. For instance, pregnant women residing in food deserts and/or food swamps experiencing food insecurity may engage deleterious health behaviors (e.g., consuming more affordable, processed foods to prevent hunger), despite the existence of federal nutrition assistance programs such as the Supplemental Nutrition Assistance Program (SNAP) and the Special Supplemental Nutrition Program for Women, Infants, and Children (WIC)—which provides supplemental food, breastfeeding and nutrition education, as well as health care and social service referrals to economically disadvantaged women (and their children ≤5 years) during prenatal and postnatal periods (56). Processed food consumption among pregnant women can exacerbate the risk for health conditions such preeclampsia (44, 45) and gestational diabetes (46), for which stark racial/ethnic inequities exist among NH-Black compared NH-White women (44–46). Considering that such inequities exist, irrespective of socioeconomic status, the social vulnerability of women from minoritized racial/ethnic groups experiencing food insecurity during pregnancy is particularly heightened. Thus, multilevel public health interventions addressing social determinants are necessary to help alleviate racial/ethnic disparities. While we were not able to produce estimates for every racial/ethnic group after stratifying by race/ethnicity due to limited sample size, the burden of food insecurity and mICVH among pregnant women from minoritized racial/ethnic groups persisted, despite similar relative associations, which warrants further investigation (41).

There are study limitations to note. First, the data from the NHIS employed a cross-sectional study design, which precludes our ability to assess causal associations. Next, due to the unavailability of data on diet in the NHIS dataset, AHA's ideal CVH metric (which includes diet) could not be used for the present study, potentially underestimating associations between food security status and mICVH among pregnant women in our results. Additionally, all data, including data on individual components of the mICVH metric were self-reported, potentially resulting in misclassification. Pregnant women belonging to historically underrepresented populations identifying as racial/ethnic groups outside of Hispanic/Latinx, NH-Black or NH-White were categorized as NH-Other, precluding our ability to make inferences across separate racial/ethnic groups. It is important for future research to disaggregate heterogenous racial/ethnic groups considering that there is evidence of differences by national origin/heritage that are overlooked when racial and ethnic groups are aggregated into broad categories. Next, alcohol consumption during pregnancy could be considered as a potential mediator that impacted our results. However, in our *post-hoc* comparison of results with and without alcohol as a confounder in our models, results were largely unchanged. Additionally, the 2020 survey year had a lower average response rate compared to previous years, likely due to the COVID-19 pandemic, introducing potential nonresponse bias among lower-income households (which would likely underestimate the magnitude of inequities in associations between food insecurity and mICVH) that cannot be eliminated (36). Further, household food security status may not capture food insecurity among the individual, also potentially producing underestimations in associations reported in our study. Also, small sample sizes among racial/ethnic groups resulted in limited power to detect associations within racial/ethnic groups. Although data were unavailable, it is worth noting that different federal nutrition assistance programs (e.g., SNAP, WIC) may moderate associations between food security status and mICVH, with WIC being particularly pertinent as it offers additional programs (e.g., breastfeeding and nutrition education, as well as health care and social service referrals to economically disadvantaged women and their children ≤5 years) catered to prenatal and postnatal care that may improve overall health (56, 57). For instance, the additional programs offered by WIC (but not SNAP) may promote both food security and mICVH (56, 57).

Our study has noteworthy strengths that contribute to the scientific literature. For example, we used a large and racially/ethnically diverse, nationally representative sample of pregnant women in the US, including individuals from historically unrepresented groups. We also included sleep (a recently established CVD risk factor) as an mICVH metric in our study to investigate associations between food insecurity and mICVH among pregnant women. Further, household food security data was collected using the USDA Family Food Supplement scale, which has been previously validated (58). Given the increased vulnerability to food insecurity among pregnant women, future studies with large samples of pregnant women (particularly those from minoritized racial/ethnic groups) investigating contributors to food insecurity and ideal CVH disparities are needed.

Given the essential role of diet for women during pregnancy, assessing household food security status in relation to mICVH during pregnancy is important. Using a modified ideal CVH metric—mICVH—inclusive of sleep, “very low/low” and “marginal” vs. “high” food security status were found to be associated with lower mICVH prevalence among pregnant women. Disparities in food insecurity prevalence and mICVH were also observed among pregnant women belonging to minoritized racial/ethnic groups, except NH-Asian adults (possibly due to racial/ethnic inequities in earnings when comparing NH-Asian and NH-White adults to NH-Black and Hispanic/Latinx adults in the US) (59). Although we were unable to estimate associations between food security status and mICVH for pregnant women by each race/ethnicity, the high burden of low food security as well as non-ideal CVH along with the association between low food security and lower prevalence of mICVH suggest racial/ethnic disparities in relationships between food insecurity and ideal CVH among pregnant women in the US (41). Considering the racial/ethnic disparities in food insecurity and mICVH, replication among diverse populations with large sample sizes is warranted. Our results may inform future studies including eventual interventions that help address food insecurity in hopes of improving CVH and addressing disparities among pregnant women.

## Data Availability

The datasets analyzed during the current study are from the National Health Interview Survey, which is publicly available, and was retrieved from https://www.cdc.gov/nchs/nhis/index.htm.

## Supplementary material

Supplementary Figure 1 Food Security Status among Pregnant Women by Race/Ethnicity, National Health Interview Survey, 2012-2018, 2020, (N=1,999). Note all estimates are weighted for the survey’s complex sampling design. Percentage may not sum to 100 due to missing values or rounding. Racial/ethnic groups for ‘NH-Other’ include women identifying as: American Indian/Alaska Native, Native Hawaiian/Pacific Islander, or multiracial.

Supplementary Figure 2 Modified Ideal Cardiovascular Health Prevalence among Pregnant Women by Food Security Status and Race/Ethnicity, National Health Interview Survey, 2012-2018, 2020, (N=1,999). Note all estimates are weighted for the survey’s complex sampling design. Percentage may not sum to 100 due to missing values or rounding. Racial/ethnic groups for ‘NH-Other’ include women identifying as: American Indian/Alaska Native, Native Hawaiian/Pacific Islander, or multiracial.

Supplementary Figure 3 Modified Ideal Cardiovascular Health Prevalence among Pregnant Women by Race/Ethnicity, National Health Interview Survey, 2012-2018, 2020, (N=1,999). Abbreviations: mICVH=Modified ideal cardiovascular health. Note all estimates are weighted for the survey’s complex sampling design. Percentage may not sum to 100 due to missing values or rounding. Racial/ethnic groups for ‘NH-Other’ include women identifying as: American Indian/Alaska Native, Native Hawaiian/Pacific Islander, or multiracial.
