# Supplementary material for: Food security status and cardiometabolic health among pregnant women in the United States
Source: Front Glob Womens Health. 2024 Feb 13;4:1286142. doi: 10.3389/fgwh.2023.1286142 (PMC10896860; doi:10.3389/fgwh.2023.1286142)
Supplement: Supplementary file 1 [file Table1.docx]

**Supplemental Table 1. Sociodemographic, Health Behavior, and Clinical Characteristics by Exclusion Criteria, National Health Interview Survey, 2012-2018, 2020, (N=140,817)**

|  | | **Included** | | **Excluded** | |
| --- | --- | --- | --- | --- | --- |
| **Characteristics** | | **n=1,999 (%)** | | **n=138,818 (%)** | |
| **Sociodemographic** | | | | | |
| Age, mean ±SE (years) | | 29.0 ±.2 | | 50.2 ±.1 | |
| 18-30 | | 62.2 | | 19.4 | |
| 31-49 | | 37.8 | | 29.4 | |
| ≥ 50 | | 0.0 | | 51.2 | |
| Missing | | 0.0 | | 0.0 | |
| Gender | | | | | |
| Women | | 100 | | 100 | |
| Missing | | 0.0 | | 0.0 | |
| Race/ethnicity | | | | | |
| Hispanic/Latinx | | 17.9 | | 13.8 | |
| NH-Asian | | 4.9 | | 5.0 | |
| NH-Black | | 15.7 | | 12.9 | |
| NH-Other | | 2.5 | | 2.2 | |
| NH-White | | 59.0 | | 65.8 | |
| Missing | | 0.0 | | 0.1 | |
| Educational Attainment | | | | | |
| < High School | | 8.8 | | 10.1 | |
| High School graduate | | 24.0 | | 26.8 | |
| Some College | | 30.4 | | 31.8 | |
| ≥ College | | 36.3 | | 30.8 | |
| Missing | | 0.6 | | 0.5 | |
| Annual household income | | | | | |
| < $35,000 | | 31.6 | | 34.9 | |
| $35-$74,999 | | 28.4 | | 27.2 | |
| ≥ $75,000 | | 35.4 | | 31.0 | |
| Missing | | 4.6 | | 6.9 | |
| Employment Status | | | | | |
| Employed | | 60.1 | | 53.7 | |
| Unemployed/not in labor force | | 39.8 | | 45.4 | |
| Missing | | 0.1 | | 0.8 | |
| Marital status | | | | | |
| Divorced/widowed | | 6.1 | | 30.4 | |
| Single/no live-in partner | | 17.7 | | 21.3 | |
| Married/living with partner/cohabitating | | 76.0 | | 47.2 | |
| Missing | | 0.1 | | 1.1 | |
| Region of residence | | | | | |
| Northeast | | 15.9 | | 17.7 | |
| Midwest | | 22.0 | | 22.5 | |
| South | | 40.5 | | 37.9 | |
| West | | 21.6 | | 21.9 | |
| Missing | | 0.0 | | 0.0 | |
| Food security status | | | | | |
| Very low | | 3.3 | | 4.7 | |
| Low | | 8.7 | | 5.9 | |
| Marginal | | 9.0 | | 7.2 | |
| High | | 79.0 | | 82.2 | |
| Missing | | 0.0 | | 0.1 | |
| **Health Behaviors** | | | | | |
| Smoking status | | | | | |
| Never/quit smoking >12 months prior | | 83.1 | | 83.7 | |
| Former | | 8.0 | | 1.2 | |
| Current | | 8.9 | | 14.3 | |
| Missing | | 0.0 | | 0.8 | |
| Alcohol consumption | | | | | |
| Current (≥1 drink past year) | | 57.3 | | 61.5 | |
| Former (no drinks past year) | | 18.3 | | 15.2 | |
| Lifetime abstinence (<12 drinks in life) | | 23.9 | | 21.6 | |
| Missing | | 0.6 | | 1.7 | |
| Leisure-time physical activity (PA) | | | | | |
| Never/unable | | 35.2 | | 33.3 | |
| Does not meet PA guidelines | | 25.6 | | 20.9 | |
| Meets PA guidelines ^b^ | | 39.3 | | 44.5 | |
| Missing | | 0.0 | | 1.3 | |
| Usual sleep duration | | | | | |
| <6 hours | | 6.8 | | 9.6 | |
| <7 hours | | 23.0 | | 30.1 | |
| 7-9 hours (recommended) | | 71.1 | | 62.7 | |
| >9 hours | | 5.9 | | 4.0 | |
| Missing | | 0.0 | | 3.2 | |
| **Clinical Characteristics** | | | | | |
| Health status | | | | | |
| Excellent/very good/good | | 95.6 | | 85.4 | |
| Fair/poor | | 4.4 | | 14.6 | |
| Missing | | 0.0 | | 0.1 | |
| Body Mass Index (BMI) | | | | | |
| Underweight (<18.5 km/m) | | 1.8 | | 2.3 | |
| Recommended (18.5-<25 kg/m) | | 39.3 | | 36.5 | |
| Overweight (25-<30 kg/m) | | 26.4 | | 27.3 | |
| Obesity (≥30 kg/m) | | 32.5 | | 29.2 | |
| Missing | | 0.0 | | 4.7 | |
| Dyslipidemia ^c^ | |  |  |  |  |
| Yes | | 2.5 | | 20.3 | |
| No | | 97.5 | | 79.7 | |
| Missing | | 0.0 | | 0.0 | |
| Hypertension ^d^ |  |  |  |  |  |
| Yes | | 8.4 | | 32.8 | |
| No | | 91.6 | | 67.1 | |
| Missing | | 0.0 | | 0.1 | |
| Diabetes or type 2 diabetes ^e^ |  |  |  |  |  |
| Yes | | 4.2 | | 16.5 | |
| No | | 95.8 | | 83.4 | |
| Missing | | 0.0 | | 0.1 | |
| Modified ideal cardiovascular health ^f^ |  |  |  |  |  |
| Yes | | 11.6 | | 9.8 | |
| No | | 88.4 | | 82.5 | |
| Missing | | 0.0 | | 7.8 | |
| Abbreviations: SE=standard error  Racial/ethnic groups for ‘NH-Other’ include women identifying as: American Indian/Alaska Native, Native Hawaiian/Pacific Islander, or multiracial. | | | | | |
| ^a^ Note all estimates are weighted for the survey’s complex sampling design. Percentage may not sum to 100 due to missing values or rounding. | | | | | |
| ^b^ Meets PA guidelines defined as ≥150 minutes/week of moderate intensity or ≥75 minutes/week of vigorous intensity or ≥150 minutes/week of moderate and vigorous intensity. | | | | | |
| ^c^ Dyslipidemia defined as currently taking prescribed medicine to lower cholesterol high cholesterol in the 12 months prior to interview. | | | | | |
| ^d^ Hypertension defined as ever told on two or more different visits that you have hypertension or high blood pressure or currently taking prescribed medicine to lower blood pressure. | | | | | |
| ^e^ Prediabetes defined as ever told by a doctor had prediabetic condition, prediabetes, or borderline diabetes. Type 2 diabetes defined as ever told by a doctor or health professional that you have diabetes or sugar diabetes and being told you have type 2 diabetes. | | | | | |
| ^f^ Modified ideal cardiovascular health includes never/quit smoking >12 months prior to interview, BMI 18.5 - <25 kg/m, meeting physical activity guidelines, sleep duration of 7-9 hours, and no dyslipidemia, hypertension, or prediabetes/type 2 diabetes | | | | | |
