# Supplementary material for: Food security status and cardiometabolic health among pregnant women in the United States
Source: Front Glob Womens Health. 2024 Feb 13;4:1286142. doi: 10.3389/fgwh.2023.1286142 (PMC10896860; doi:10.3389/fgwh.2023.1286142)
Supplement: Supplementary file 2 [file Table2.docx]

# Supplemental Table 2. U.S. Food Security Survey Module, Family Food Supplement, United States Department of Agriculture 2012

| **Item** | **Response** |
| --- | --- |
| 1. Family worried food would run out before got money to buy more, last 30 days | 1. Often true 2. Sometimes true 3. Never true 4. Don’t know |
| 2. Food did not last until family had money to get more, last 30 days | 1. Often true 2. Sometimes true 3. Never true 4. Don’t know |
| 3. Family could not afford to eat balanced meals, last 30 days | 1. Often true 2. Sometimes true 3. Never true 4. Don’t know |
| 4. Family members cut size or skipped meals because not enough money, last 30 days | 1. Yes 2. No 3. Don’t know |
| 4a. Number of days any family members cut size or skipped meals for financial reasons, last 30 days | 1. 0 to 30 2. Don’t know |
| 5. Ever ate less than felt should because not enough money, last 30 days | 1. Often true 2. Sometimes true 3. Never true 4. Don’t know |
| 6. Ever hungry but did not eat because not enough money, last 30 days | 1. Often true 2. Sometimes true 3. Never true 4. Don’t know |
| 7. Ever lost weight because not enough money for food, last 30 days | 1. Often true 2. Sometimes true 3. Never true 4. Don’t know |
| 8. Any family members not eat for a whole day because not enough money for food, last 30 days | 1. Yes 2. No 3. Don’t know |
| 8a. Number of days any family member did not eat due to lack of money, last 30 days | 1. 0 to 30 2. Don’t know |
