# Supplementary material for: Food security status and cardiometabolic health among pregnant women in the United States
Source: Front Glob Womens Health. 2024 Feb 13;4:1286142. doi: 10.3389/fgwh.2023.1286142 (PMC10896860; doi:10.3389/fgwh.2023.1286142)
Supplement: Supplementary Figure 3 — Modified Ideal Cardiovascular Health Prevalence among Pregnant Women by Race/Ethnicity, National Health Interview Survey, 2012-2018, 2020, (N=1,999). Abbreviations: mICVH=Modified ideal cardiovascular health. Note all estimates are weighted for the survey’s complex sampling design. Percentage may not sum to 100 due to missing values or rounding. Racial/ethnic groups for ‘NH-Other’ include women identifying as: American Indian/Alaska Native, Native Hawaiian/Pacific Islander, or multiracial. [file Image3.pdf]

Supplemental Figure 3. Modified Ideal Cardiovascular Health Prevalence among Pregnant Women by Race/Ethnicity, National Health Interview Survey, 2012-2018, 2020, (N=1,999) [a]

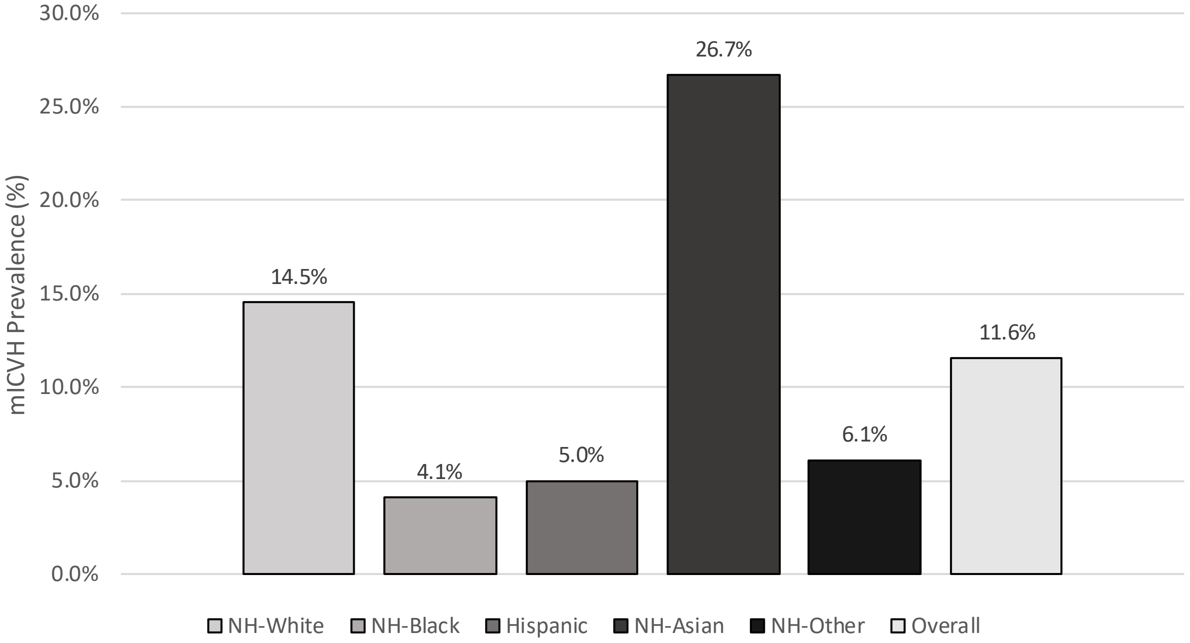

Abbreviations: mICVH = Modified ideal cardiovascular health

[a] Note all estimates are weighted for the survey's complex sampling design. Percentage may not sum to 100 due to missing values or rounding. Racial/ethnic groups for 'NH-Other' include women identifying as: American Indian/Alaska Native, Native Hawaiian/Pacific Islander, or multiracial.
